# Identification of Prognostic Signature and Gliclazide as Candidate Drugs in Lung Adenocarcinoma

**DOI:** 10.3389/fonc.2021.665276

**Published:** 2021-06-24

**Authors:** Yang Cheng, Kezuo Hou, Yizhe Wang, Yang Chen, Xueying Zheng, Jianfei Qi, Bowen Yang, Shiying Tang, Xu Han, Dongyao Shi, Ximing Wang, Yunpeng Liu, Xuejun Hu, Xiaofang Che

**Affiliations:** ^1^ Department of Respiratory and Infectious Disease of Geriatrics, The First Hospital of China Medical University, Shenyang, China; ^2^ Key Laboratory of Anticancer Drugs and Biotherapy of Liaoning Province, The First Hospital of China Medical University, Shenyang, China; ^3^ Department of Medical Oncology, The First Hospital of China Medical University, Shenyang, China; ^4^ Liaoning Province Clinical Research Center for Cancer, The First Hospital of China Medical University, Shenyang, China; ^5^ Marlene and Stewart Greenebaum Comprehensive Cancer Center, University of Maryland, Baltimore, MD, United States

**Keywords:** lung adenocarcinoma, prognosis, signature, drug repositioning, gliclazide

## Abstract

**Background:**

Lung adenocarcinoma (LUAD) is the most common pathological type of lung cancer, with high incidence and mortality. To improve the curative effect and prolong the survival of patients, it is necessary to find new biomarkers to accurately predict the prognosis of patients and explore new strategy to treat high-risk LUAD.

**Methods:**

A comprehensive genome-wide profiling analysis was conducted using a retrospective pool of LUAD patient data from the previous datasets of Gene Expression Omnibus (GEO) including GSE18842, GSE19188, GSE40791 and GSE50081 and The Cancer Genome Atlas (TCGA). Differential gene analysis and Cox proportional hazard model were used to identify differentially expressed genes with survival significance as candidate prognostic genes. The Kaplan–Meier with log-rank test was used to assess survival difference. A risk score model was developed and validated using TCGA-LUAD and GSE50081. Additionally, The Connectivity Map (CMAP) was used to predict drugs for the treatment of LUAD. The anti-cancer effect and mechanism of its candidate drugs were studied in LUAD cell lines.

**Results:**

We identified a 5-gene signature (KIF20A, KLF4, KRT6A, LIFR and RGS13). Risk Score (RS) based on 5-gene signature was significantly associated with overall survival (OS). Nomogram combining RS with clinical pathology parameters could potently predict the prognosis of patients with LUAD. Moreover, gliclazide was identified as a candidate drug for the treatment of high-RS LUAD. Finally, gliclazide was shown to induce cell cycle arrest and apoptosis in LUAD cells possibly by targeting CCNB1, CCNB2, CDK1 and AURKA.

**Conclusion:**

This study identified a 5-gene signature that can predict the prognosis of patients with LUAD, and Gliclazide as a potential therapeutic drug for LUAD. It provides a new direction for the prognosis and treatment of patients with LUAD.

## Introduction

Lung cancer is the most common malignant tumor with the highest morbidity and mortality worldwide, including China (1). Non-small cell lung cancer (NSCLC) accounts for about 85% of newly diagnosed cases, of which lung adenocarcinoma (LUAD) is the most common subtype (2, 3). Although the therapeutic approaches of LUAD, such as surgery, tyrosine kinase inhibitors (TKIs), immunotherapy and individualized therapy strategy, have been greatly improved and widely applied in clinic practice, the prognosis is not optimistic with only 16% of the 5-year overall survival rate (4–6). Therefore, it is necessary to accurately predict the prognosis and give the best treatment to improve the curative effect and prolong the patient survival in patients with LUAD.

The biological characteristics of LUAD including poor differentiation, high malignancy and more aggressiveness, are known to associate with unfavorable prognosis. However, prognostic prediction solely based on these pathological characteristics is of limited efficiency and accuracy (7, 8). The combination of prognostic biomarkers and pathological characteristics is helpful to improve the ability to predict prognosis. Currently, with the development of microarray analysis and whole genome sequencing, many studies have been carried out to screen and mine the prognostic markers in cancers including NSCLC. For example, Zuo et al. identified a robust six-gene prognostic biomarkers to predict disease-free survival (DFS) and overall survival (OS) for NSCLC (9); He et al. digged out a reliable 8-gene prognostic biomarker for early NSCLC (10). However, these biomarkers are rarely successfully used in clinical practice due to the lack of experimental and clinical verification. Therefore, it is of great significance to develop new biomarkers to improve the accuracy of prognostic prediction for LUAD patients.

Moreover, due to genetic heterogeneity, appropriate treatment for patients with different genetic characteristics is also important for the prolongation of prognosis. However, most patients lose effective treatment because of primary or secondary drug resistance (11). Therefore, it is necessary to find new treatment strategies for the high-risk groups. Connectivity Map (CMAP), as the database developed by Broad Research Institute to explore the functional relationship between small molecule compounds, genes expression change and disease states, can be used to identify potential therapeutic drugs according to the expression characteristics of up-regulated or down-regulated genes at the genomic level (12–14). Screening small molecular drugs used in clinic, which is also called conventional drug in new use, can avoid the time-consuming and expensive procedure of new drug development (15, 16). Therefore, in-depth understanding of LUAD expression profile and other biological information will facilitate the screening of effective drugs and improve of LUAD prognosis.

In this study, we combined multiple gene expression data sets to develop and verify a five-gene signature risk model, which can accurately evaluate individual prognosis in patients with LUAD. In addition, we combined the survival-related differentially expressed genes (DEGs) of prognostic significance with CMAP to find and verify gliclazide as a potential drug therapy for lung cancer *in vitro* for the first time. Therefore, our research provides a new strategy for the prognosis and treatment of lung adenocarcinoma.

## Materials and Methods

### Data Source and Preprocessing

The mRNA expression profiles and clinical data (including 535 tumor samples and 59 normal samples) of LUAD were downloaded from TCGA database (http://ualcan.path.uab.edu/cgi-bin/ualcan-res.pl) on the 9th December, 2019. LUAD-related datasets GSE18842, GSE19188, GSE40791 and GSE50081, downloaded from the Gene Expression Omnibus (GEO) database (https://www.ncbi.nlm.nih.gov/geo/), were used as validation sets. For data cleaning, samples with missing clinical data were excluded.

### DEGs Screening

The merging of three GEO datasets (GSE18842, GSE19188 and GSE40791) was accomplished by using dplyr package in R (17). The sva package was eliminated batch effects and other unrelated variables in high-throughput experiments (11). DEGs between tumor- and normal- samples were identified by Limma package in R (18). |log2 fold-change (FC)| >2, P <0.05 were used as the cut-off values of DEGs.

### Prognostic Gene Signature Screening

To screen the prognostic related genes, DEGs were analyzed by univariate Cox regression analysis and P <0.05 was used as cutoff in TCGA-LUAD. In order to reduce the complexity and multicollinearity of the model, the “glmnet” R package was used for Lasso regression analysis (19), and the stepwise multiple Cox regression method was used to construct the optimal model. Then, based on the linear combination of the expression levels and the weighted regression coefficient obtained by multiple Cox regression method, the prognosis-related risk score was established. Risk score *(RS) = expression of gene_1_  ×  β_1_ + expression of gene_2_  ×  β_2_ +⋯+ expression of gene_n_  ×  β_n_* (20). According to the median of RS, the patients were divided into low-RS (low risk) group and high-RS (high risk) group. Prognostic cancer cohort GSE50081 used the same formula as the coefficient in TCGA-LUAD to obtain the corresponding RS and was used as an independent validation dataset.

### Gene Set Enrichment Analysis

To explore the prognostic biomarker involved potential biological processes. GSEA analysis was conducted by expression of 25,331 genes from TCGA samples in GSEA v4.0.3 (21). As the classical gene set in Molecular Signatures Database (MSigDB), “c2.cp.kegg.v7.0.symbols.gmt (Curated)” was considered. P <0.05 and a false discovery rate (FDR) of q <0.25 was regarded as the reference value.

### CMAP Analysis

CMAP_(http://www.broad.mit.edu/cmap/)_ provides a wealth of information about small molecular drugs, gene expression and diseases that are closely interrelated at the genomic level. Therefore, researchers can link gene expression data to disease-related drugs. In order to find new drug candidates for LUAD patients, the DEGs related to survival were uploaded to CMAP, and a corresponding analysis result could be obtained after passed the Kolmogorov–Smirnov test. The drugs with a negative score indicating their potential of anti-tumor effect were selected as the new target drug candidates for LUAD patients with high-RS.

### Homologous Modeling and Molecular Docking

The molecular structure of gliclazide was obtained from PubChem Compound (https://pubchem.ncbi.nlm.nih.gov/). The 3D coordinates of CCNB1 (PDB ID, 6GU2; resolution, 2.0 Å) and CDK1 (PDB ID, 6GU2; resolution, 2.0 Å) and AURKA (PDB ID, 5L8J; resolution, 1.68 Å) were downloaded from the PDB (http://www.rcsb.org/pdb/home/home.do).The CCNB2 of amino acids sequences were analyzed by EXpasy (http://swissmodel.expasy.org/) for lacking of its complete crystal structure. Ramachandran plots were used to assess stereo-chemical quality with the default parameters. Auto dock Vina 1.1.2 (http://autodock.scripps.edu/) and Pymol software 2.3 (DeLano Scientific, Portland, USA) were used for molecular docking studies and model visualization, respectively (11, 22).

### Compounds and Cell Culture

Gliclazide (Sigma Aldrich) was dissolved with dimethyl sulfoxide (DMSO) to a 500 mM storage concentration and stored at −20°C. The concentration of DMSO was kept less than 0.4% v/v throughout each experiment. Two human LUAD cell lines A549 and H1299 were purchased from Shanghai Institutes for Biological Sciences, Chinese Academy of Sciences (Shanghai, China). Cells were cultured in RPMI1640 medium supplemented with 10% fetal bovine serum at 37°C in a humidified atmosphere of 5% CO_2_.

### MTS Assay

Cells with density of 3,000 cells/well in 96-well plates were treated with various concentrations of Gliclazide (0, 500, 1,000 and 2,000 μM) for 24, 48 and 72 h, and then the cell-viability assay (MTS assay; Promega, Madison WI) was carried out according to the reagent’s instructions.

### Colony Formation Assay

A549 or H1299 cells (400 cells/well) were seeded into 12-well plate. Next day, cells were treated with various concentrations (0, 500, 1,000 and 2,000 μM) of Gliclazide and continuously incubated for 10–14 days. After cells were stained with crystal violet, colonies were counted using ImageJ software (NIH, Bethesda, MD).

### Cell Cycle Analysis

A549 or H1299 cells were treated with different concentrations of Gliclazide (0, 500, 1,000 and 2,000 μM) for 48 h. Then, cells were harvested and fixed with 70% (v/v) cold ethanol at 4°C overnight. After 30 min-incubation with 100 μg/ml RNase A and 10 μg/ml propidium iodide (PI) staining solution in dark, cells were analyzed by FACScan flow cytometer (Becton Dickinson, USA) and evaluated using the ModFit program software.

### Western Blot Analysis

A549 or H1299 cells were treated with different concentrations of Gliclazide (0, 500, 1,000 and 2,000 μM) for 72 h. Then, total protein was extracted for western blot as our previous studies (23). The following antibodies were used in western blot analyses: anti-cyclin A (SANTA, 1:1,000), anti-cyclin B1 (SANTA, 1:1,000), anti-cyclin D1 (SANTA, 1:1,000), anti-cyclin E (SANTA, 1:1,000), anti-GAPDH (SANTA, 1:5,000) and anti-PARP (CST, 1:1,000).

### Statistical Analysis

Kaplan–Meier curves were drawn and the significant difference was checked by log-rank test. The Receiver Operating Characteristic (ROC) analysis was used to detect the sensitivity and specificity of the risk score in predicting survival. The area under the ROC curve (AUC) was used to evaluate the efficiency of prognosis (11, 24). X^2^ test was used to evaluate the correlation of RS with clinical characteristics. Univariate and multivariate Cox proportional hazard regression analysis was also performed to access the relationship between RS and OS. Based on the multivariate Cox analysis, a nomogram was constructed with the “rms” package in R (20, 25). A P-value of less than 0.05 was set as statistically significant for all the analyses. All statistical analyses were performed using R version 3.6.3 (http://www.R-project.org), SPSS 16.0 and Graph Pad Prism 7 software.

## Results

### Construction and Assessment of a Five-Gene Prognostic Signature

**Figure 1:**
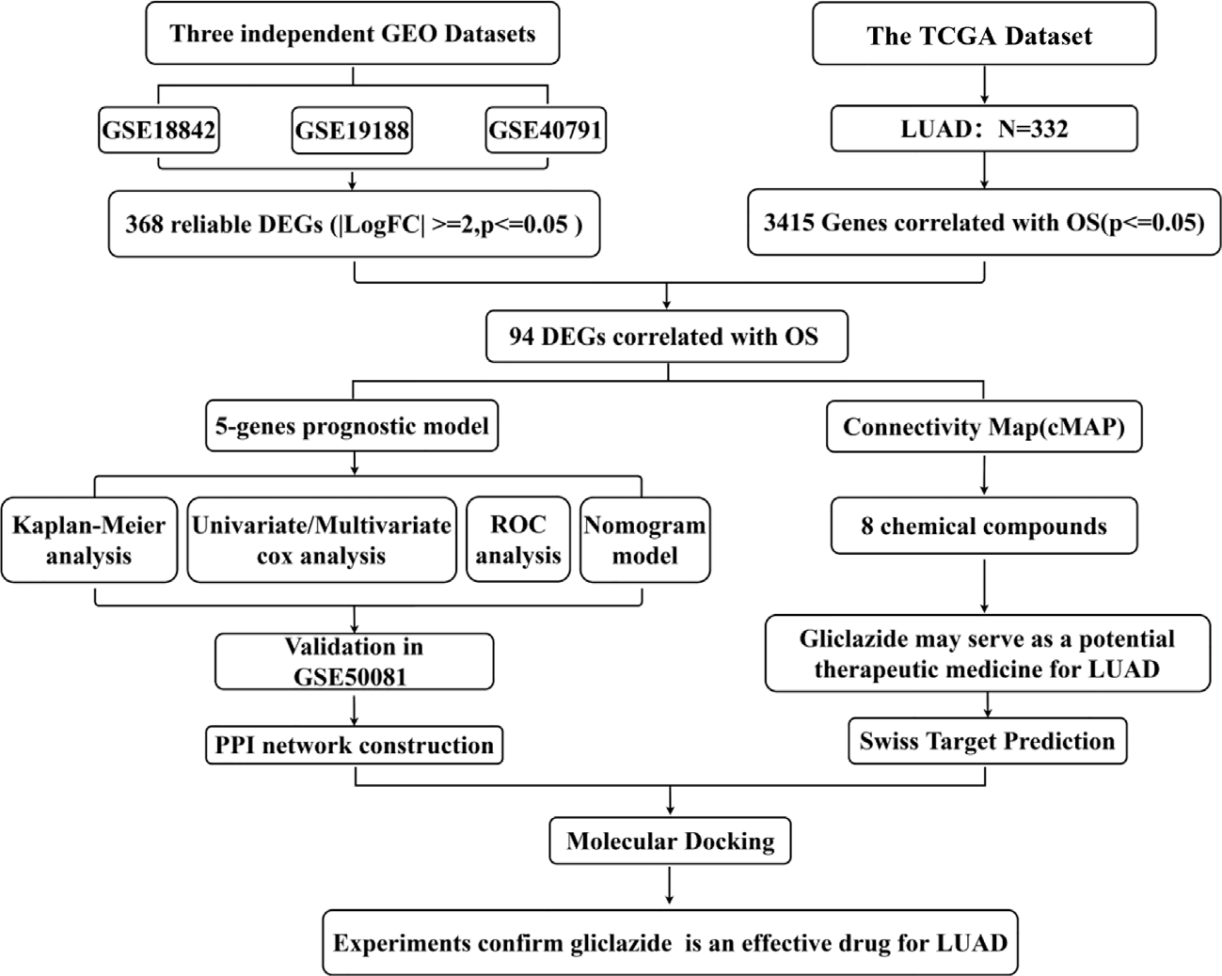
Study flow diagram. OS, overall survival; DEGs, differentially expressed genes.

**Figure 2:**
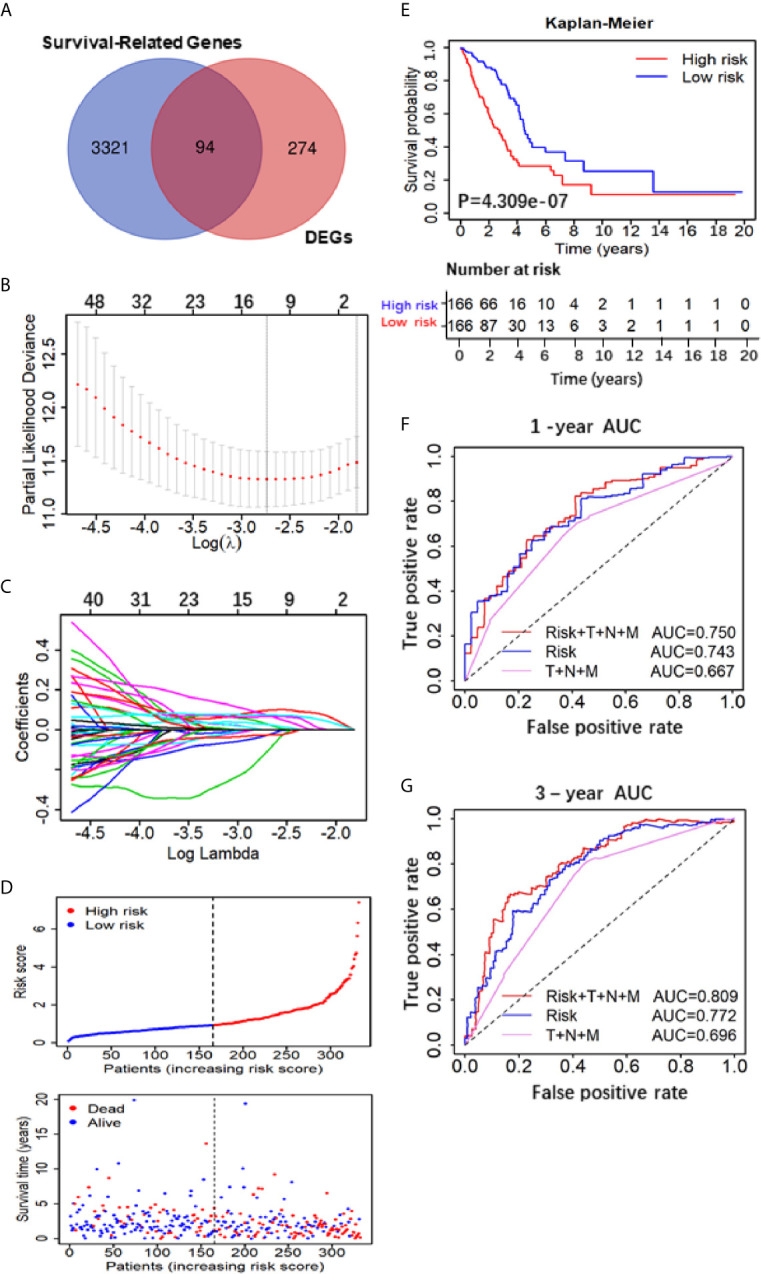
Construction and assessment of the Five-Gene Prognostic Signature. **(A)** Venn diagram depicting potential prognostic gene of the inter section between DEGs and Survival-Related Genes; **(B)** A coefficient profile plot was generated against the log (lambda) sequence. Selection of the optimal parameter (lambda) in the LASSO model. **(C)** LASSO coefficient profiles of the nine candidates in TCGA training set. **(D)** Patients’ survival status distribution by the risk score; patient survival status distribution of the low-risk group and the high-risk group; **(E)** Kaplan–Meier curves for the low- and high-RS groups; **(F**, **G)** the receiver operating characteristic (ROC) curve validation of prognostic value by the risk score of 1 and 3 years.

The flow chart of this study was shown in **Figure 1**, and details of the datasets used in this study were shown in **Table 1**. To screen out the genes with prognostic prediction value for LUAD, we first performed the univariate Cox regression analyses on TCGA-LUAD dataset including 332 patients with complete information, and found that 3,516 genes were significantly associated with overall survival (OS) (P <0.05). Then, using a total of 210 tumor samples and 231 non-tumor samples integrated from GSE18842, GSE19188 and GSE40791 datasets, we analyzed DEGs with the criteria of P <0.05 and |log2 fold-change (FC)|>2, and 368 DEGs including 103 up-regulated and 265 down-regulated genes were screened out. Among them, 94 genes were overlapped with the TCGA result of univariate Cox regression analyses, including 54 up-regulated genes and 40 down-regulated genes (**Figure 2A**). Thus, these 94 genes were regarded as prognostic gene candidates. Next, we performed Lasso-penalized Cox analysis with cross-validation to pick out 9 genes from the 94 candidates (**Figures 2B, C** and **Table S1**).We further performed a stepwise multivariate Cox regression analysis to finally identify five independent prognostic genes. And we verified the five genes in TCGA-LUAD, and the results were consistent with the original results, in which the expression of KRT6A and KIF20A was higher in cancer tissues than that in paracancerous tissues, while the expression of KLF4, LIFR and RGS13 is on the contrary (**Supplementary Figures 1A–E**). Furthermore, among the five genes, KIF20A, KLF4 and KRT6A were prognostic risk factors (HR >1), whereas LIFR and RGS13 were prognostic protective factors (HR <1) (**Table 2**).

**Table 1:** Characteristics of the public microarray datasets used in this study.

Study	Species/array platform	Samples	Type	Function
TCGA-LUAD	Human Illumina HiSeq 2000	332 Cancer	LUAD	Training set
GSE50081	(GPL570)	128 Cancer	LUAD	Validation set
GSE18842	(GPL570)	45 Normal and 46 Cancer	NSCLC	For DEGs
GSE19188	(GPL570)	65 Normal and 91 Cancer	NSCLC	For DEGs
GSE40791	(GPL570)	100 Normal and 94 Cancer	NSCLC	For DEGs

**Table 2:** Multivariate Cox regression analysis of the 5-gene signature.

ID	Coef	HR	HR.95L	HR.95H	P-value
KIF20A	0.301291283	1.351602983	1.103810884	1.65502139	0.003547525
LIFR	−0.182264632	0.833380772	0.672709557	1.032427002	0.095329705
RGS13	−1.100168875	0.332814875	0.117059758	0.946232446	0.039052759
KLF4	0.240230298	1.27154195	1.070939257	1.509720483	0.006100447
KRT6A	0.085854478	1.089647749	1.012552387	1.172613123	0.0218395

HR, hazard ratio (HR >1, risk factor; HR <1, Protective factors); 95% CI, 95%confidence interval.

To evaluate the 5-gene prognostic signature, we calculated the risk score (RS) of each sample in TCGA-LUAD according to the expression levels of five genes weighted by their relative coefficient using the following formula: RS ***=*** *(0.3013 × KIF20Aexp) + (−0.1823 × LIFRexp) + (−1.100 × RGS13exp) + (0.2402 × KLF4exp) + (0.0859 × KRT6Aexp)* (**Table 2**). Then, we separated the patients into high-RS and low-RS groups according to the median of RS (**Figure 2D**), and compared the OS using Kaplan–Meier analysis. The results showed that patients in high-RS group exhibited significantly shorter OS than those in low-RS groups (P = 4.309e*−*07, **Figure 2E**). Moreover, we evaluated the sensitivity and specificity of RS for OS prediction using a time-dependent ROC curve at 1- and 3-year. Notably, AUC values of RS achieved 0.743 and 0.722 at 1- and 3-year (**Figures 2F, G**), respectively. They were significantly better than the AUC of TNM alone, but slightly lower than the AUC of RS and TNM combination, indicating the good sensitivity and specificity of RS based on 5-gene signature.

To further verify the prognostic predictive value of 5-gene signature, we selected GSE50081 dataset including 128 LUAD patients as the validation set (**Table S2**). The survival trend of validation dataset was highly similar to that of TCGA-LUAD dataset in Kaplan–Meier analysis, and the ROC curve proved the accuracy of prognostic prediction, thereby further supporting that the high-RS value indicated a poor prognosis (**Figures 4A–D**). Together, these results above demonstrate that the 5-gene signature is credible and effective for prognostic prediction in LUAD.

### Validation of the Five-Gene Prognostic Signature

**Figure 3:**
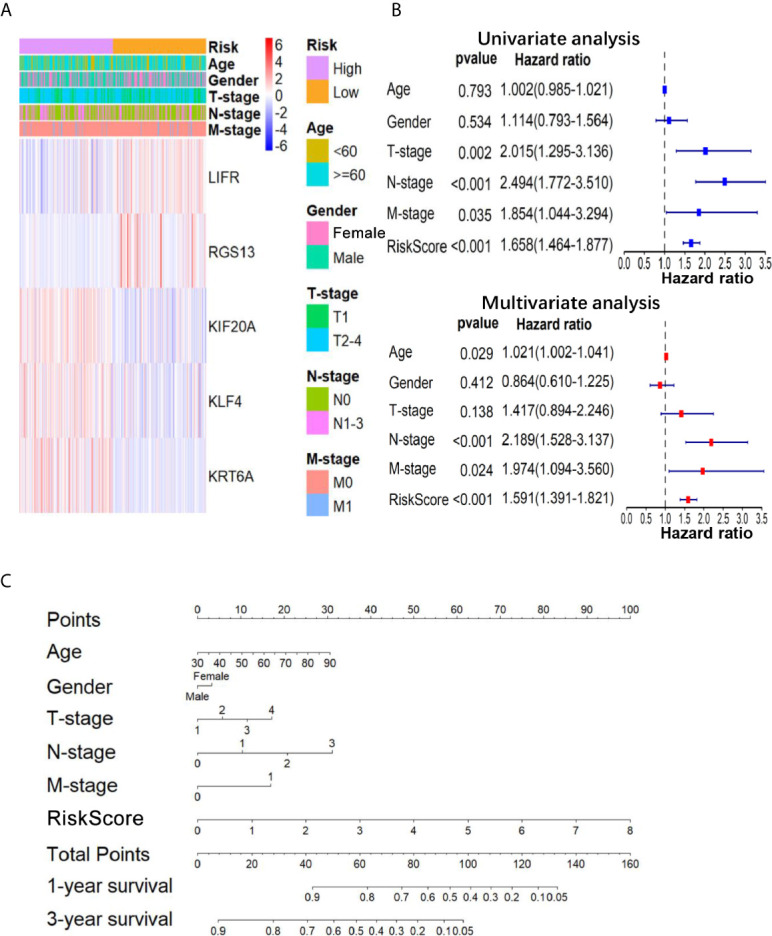
The relationship between the 5-gene based RS and clinical information. **(A)** The heatmap showed the expression levels of the 5-genes in the low- and high-RS groups; The distribution of clinicopathological features was compared in low- and high-RS groups. **(B)** The univariate and multivariate Cox analysis for the independent 5-gene signature; **(C)** a nomogram was used to predict the overall survival at 1 year and 3 years with RS and clinical information.

**Figure 4:**
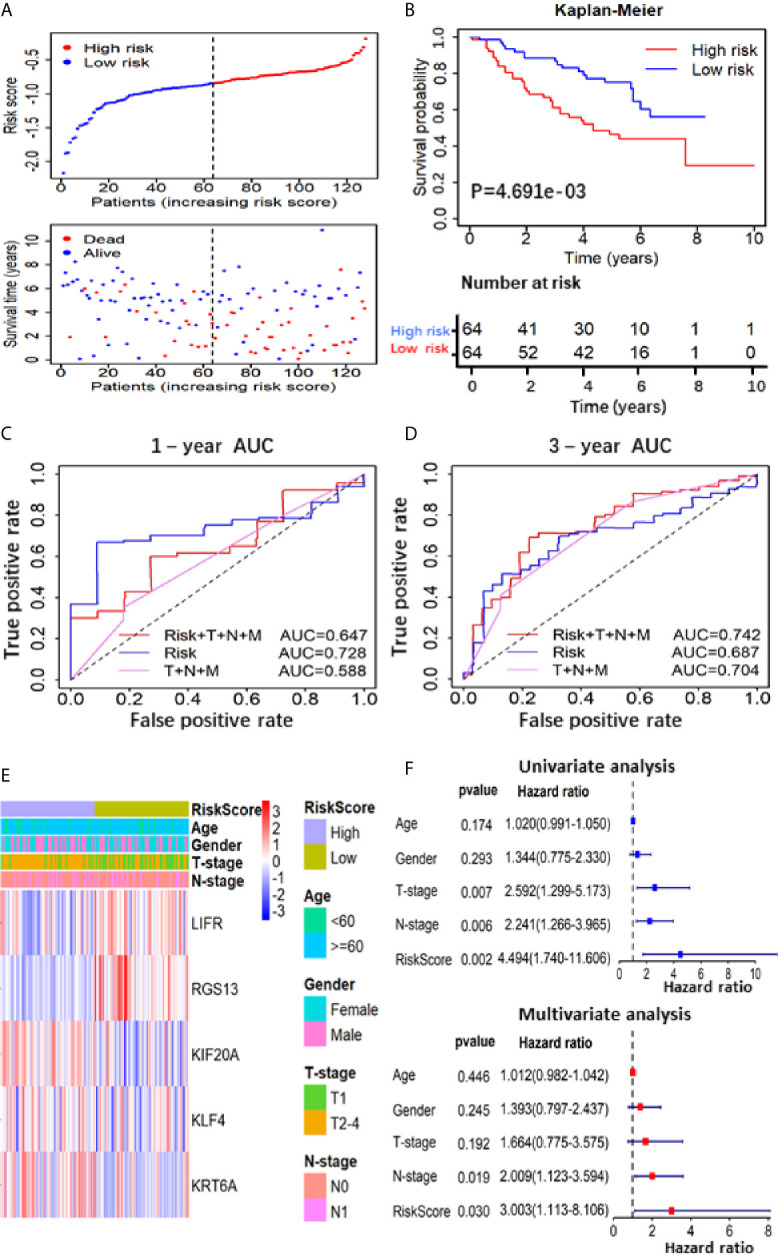
Prognostic analysis based on of the 5 gene-RS model in GSE50081 validation cohort. **(A)** Patients’ survival status distribution by the risk score; patient survival status distribution of the low-RS group and the high-RS group; **(B)** Kaplan–Meier curves for the low- and high-RS groups; **(C**, **D)** the receiver operating characteristic (ROC) curve validation of prognostic value by the risk score of 1 and 3 years; **(E)** The heatmap showed the expression levels of the 5-genes and the distribution of clinicopathological features in the low- and high-RS groups; **(F)** The univariate and multivariate Cox analysis for the independent 5-gene signature.

To further confirm the important role of 5-gene signature in LUAD, we analyzed the correlation of RS with clinicopathological parameters. The heatmap in **Figure 3A** showed the expression pattern of five genes in high- and low- LUAD groups of TCGA cohorts. Three risk genes KIF20A, KLF4 and KRT6A were highly expressed in the high-RS group, whereas protective genes LIFR and RGS13 were highly expressed in low-RS group (**Figure 3A**). The correlation analysis result showed that RS was significantly associated with T-stage, N-stage in TCGA-LUAD cohorts (**Table S2** and **Figure 3B**). Subsequently, LUAD patients were divided into subgroups according to age, gender and TNM-stage, respectively, and KM analysis was further performed in each subgroup. The results showed that in subgroup of age >65, male and T1/2, the OS of patients with high-risk was significantly shorter than that with low-risk (P <0.05, **Figures S1F–R**). Moreover, univariate and multivariate Cox regression model suggested that RS in TCGA-LUAD was significantly associated with overall survival (OS) (HR =1.658, 95% CI =1.464-1.877, P <0.001 for univariate model; HR =1.591, 95% CI =1.391-1.891, P <0.001 for multivariate model). In order to keep the format consistent, we have added or deleted the corresponding spaces, which have been modified as above (**Figure 3B** and **Table S4**). Similar results were obtained from GSE50081 cohorts (**Figures 4E, F** and **Table S2**). Furthermore, we constructed a nomogram for 1- and 3-year OS prediction by integrating both 5-gene signature and conventional clinicopathological factors (**Figure 3C**). The C-index of 0.725 indicated the good performance of prediction model. Thus, the 5-gene signature is valid and reliable for prognostic prediction in LUAD.

### Gene Set Enrichment Analysis

**Figure 5:**
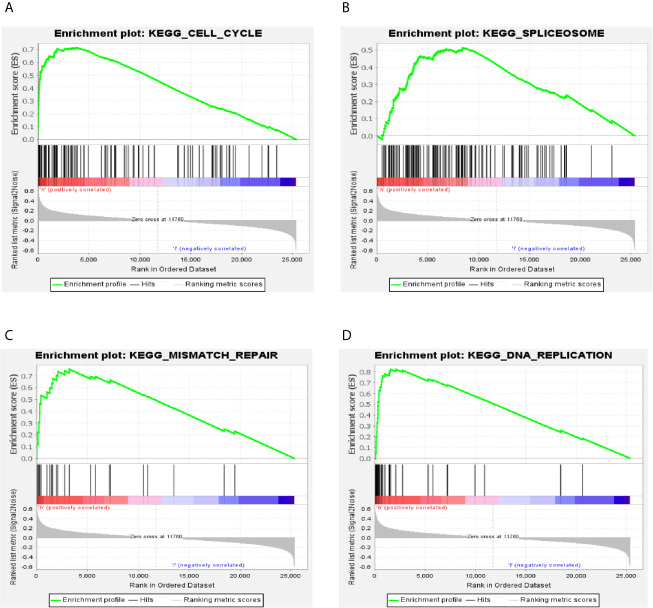
GSEA analysis of the differentially expressed genes between high- and low-RS groups. **(A)** Cell cycle; **(B)** spliceosome; **(C)** mismatch repair; **(D)** DNA replication.

To identify the significant changes of biological pathways between high- and low-RS groups, the GSEA was performed. Based on the cut-off criteria of FDR <0.25 and P <0.05, 18 significantly altered pathways were selected, including “cell cycle”, “spliceosome”, “DNA replication”, “mismatch repair” and other pathways (**Figures 5A–D** and **Table S4**). This result exhibited a strong connection between the identified signature and tumor growth, indicating 5-gene signature might lead to poor prognosis by promoting cell proliferation and DNA damage repair in LUAD.

### Screening of Potential Drugs for LUAD by CMAP Analysis

**Figure 6:**
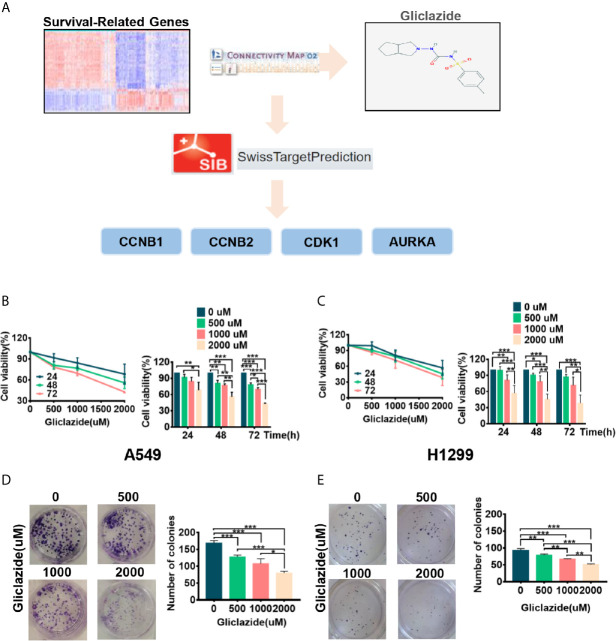
The screening process and proliferation experiment verification of gliclazide. **(A)** The methodology used in the compilation of survival-differentially expressed genes and the CMAP algorithm. **(B–E)** A549 and H1299 cells were treated with gliclazide for 24, 48 and 72 h and the cell viability was measured by MTT Assay **(B**, **C)**, or colony-forming assay **(D**, **E)**. One-way ANOVA was used for statistical analyses. Data are plotted as mean ± SD. P values are labeled in the figures. *P < 0.05; **P < 0.01; ***P < 0.001.

To identify novel drugs targeting LUAD patients with poor OS, we performed CMAP analysis on the 94 prognostic gene candidates. We searched for negatively-correlated gene expression patterns associated with drug-treated cancer cells in the CMAP database using the cut-off criteria of percent non-null ≥75 and mean ≤*−*0.4. The analyses screened out eight drug candidates, which were not reported to play anti-LUAD effect before (**Table 3**). Considering the clinical superiority, we selected gliclazide to validate its anti-cancer effect and molecular mechanism in LUAD (**Figure 6A**).

**Table 3:** Eight candidate drugs of connectivity map analysis.

CMAP name	Mean	P	Percent non-null
medrysone	−0.68	0.00064	100
0175029-0000	−0.649	0.00073	100
ginkgolide A	−0.73	0.00105	100
repaglinide	−0.722	0.00275	100
trioxysalen	−0.663	0.00336	100
gliclazide	−0.66	0.00432	100
0173570-0000	−0.734	0.00465	100
eucatropine	−0.621	0.00528	100
0297417-0002B	−0.658	0.00863	100

### Gliclazide Restrains the Proliferation of Lung Adenocarcinoma Cells

To experimentally validate the therapeutic efficacy of gliclazide, we assessed the effect of gliclazide on LUAD cell lines, A549 and H1299. Gliclazide treatment significantly inhibited the growth of two LUAD cells in a dose- and time-dependent manner (**Figures 6B, C** **)**. Similar results were obtained by colony formation assay (**Figures 6D, E** **)**. Hence, these findings supported an anti-cancer effect of gliclazide by inhibiting the proliferation of LUAD cells.

### Identification of the Candidate Targets of Gliclazide by Molecular Docking Analysis

**Figure 7:**
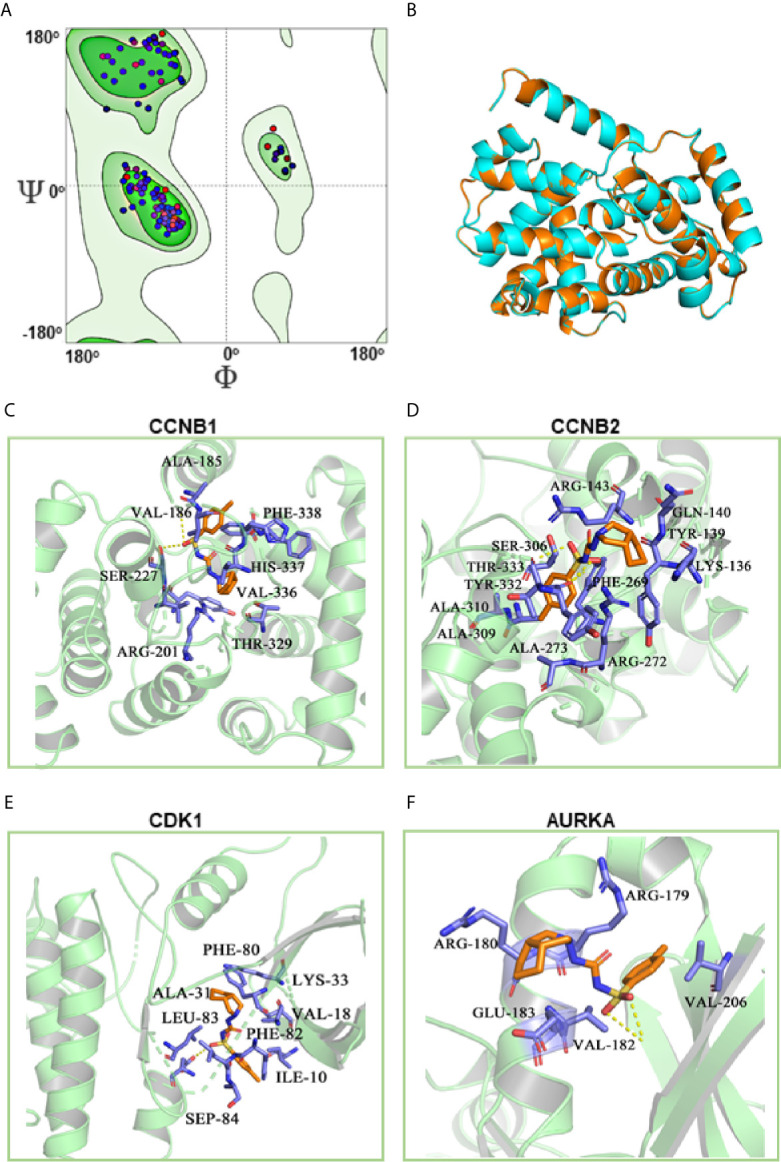
Molecular docking of gliclazide targets. The results of CCNB2 homology modeling. Ramachandran plot analysis showed that existence of 98.4% of all residues in the allowed regions for CCNB2 **(A)**, the structure of CCNB2 was basically the same as that of template protein, and the identity of their amino acid sequence was 64.5%, highlighting the accuracy of the predicted structures **(B)**. **(C–F)** Molecular docking analyses for Gliclazide with target proteins.

**Figure 8:**
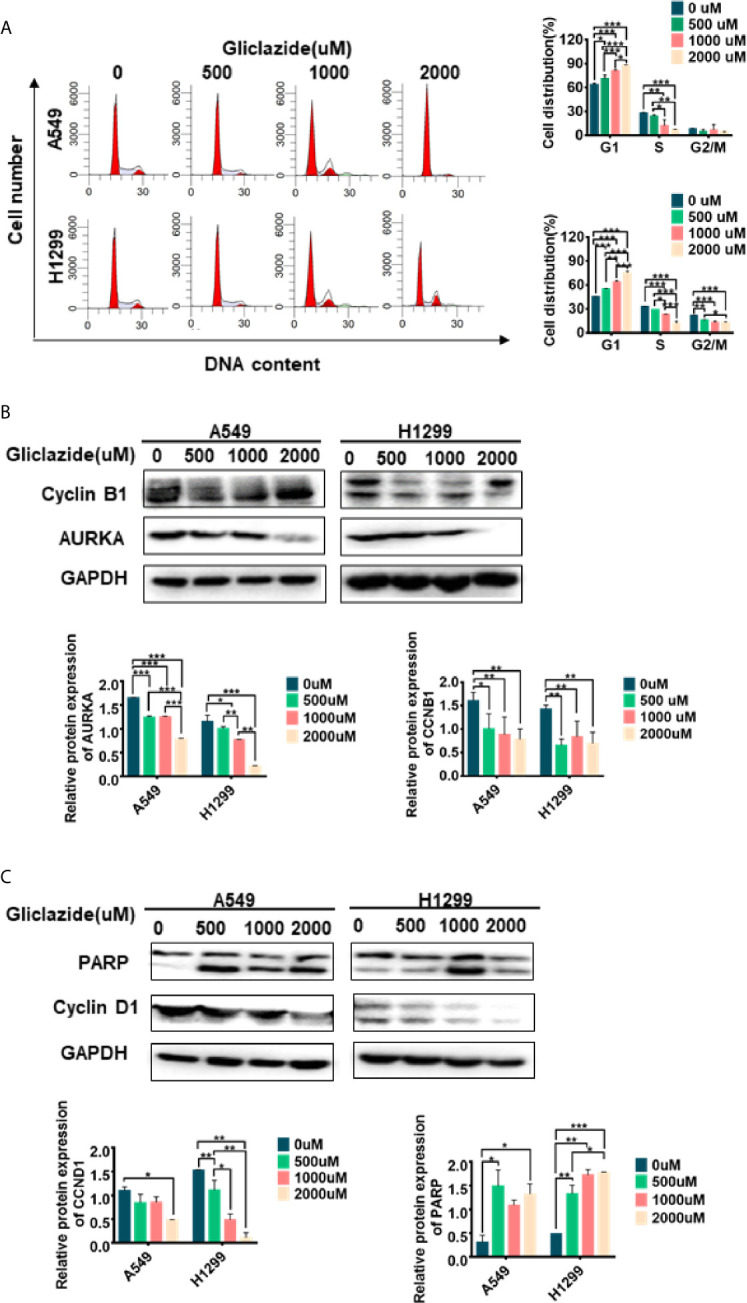
Experimental verification of Gliclazide on cell cycle and apoptosis. **(A)** Effect of the gliclazide on cell cycle distribution of A549 and H1299 cells exposed to gliclazide for 48 h. Histograms of cellular DNA content obtained by flow cytometry have been represented. **(B**, **C)** Protein expression of CyclinD1, cyclin B1, AURKA and PARP were quantified *via* western blotting, with GAPDH as the loading control. One-way ANOVA was used for statistical analyses. Data are plotted as mean ± SD. P values are labeled in the figures. *P < 0.05; **P < 0.01; ***P < 0.001.

To clarify the molecular mechanism of gliclazide in inhibiting LUAD cells, we used SwissTargetPrediction online database(http://www.swisstargetprediction.ch/) to predict the key targets of gliclazide. The analyses screened out four potential target candidates including CCNB1, CCNB2, CDK1 and AURKA (**Figure 6A**). Then, we performed the molecular docking analysis to evaluate the binding of gliclazide to the four targets. Molecular docking analysis was based on the crystal structure of proteins and structure of drugs. The complete crystal structures of CCNB1, CDK1 and AURKA, but not CCNB2, were available. Sowe first constructed the homologous modeling of CCNB2 using the online model prediction website Swiss-Model (https://swissmodel.expasy.org/interactive/). Ramachandran diagram and other analysis showed that the constructed CCNB2 structure is accurate and reasonable and can be used for the next step of molecular docking (**Figures 7A, B** **)**. Next, the binding poses and interactions of 4 drug targets with gliclazide were obtained with Auto dock Vina v.1.1.2 and the binding energy for each interaction was generated with no obstacles. The results showed that gliclazide bound to CCNB1, CCNB2, CDK1 and AURKA through visible hydrogen bonds and strong electrostatic interactions, and the hydrophobic pocket of each target was occupied successfully by gliclazide with the low binding energy of *−*8.9, *−*8.6, *−*8.3 and *−*6.9 kcal/mol, respectively (**Table 4**, **Figures 7C–F**), indicating the highly stable binding. Moreover, we selected the target protein with the highest binding capacity, CCNB1, and the lowest binding capacity, AURKA, to verify the above analysis results in A549 and H1299 cells. As we expected, 72h-treatment of gliclazide did significantly inhibit the protein expression of CCNB1 and AURKA (**Figure 8B**), supporting the results of online data analysis and molecular docking.

**Table 4:** Binding energy for targets with their drugs.

Protein	Docking score (kcal/mol)
	Gliclazide
CCNB1	−8.9
CCNB2	−8.6
CDK1	−8.3
AURKA	−6.9

### Gliclazide Induces Cell Cycle Arrest and Apoptosis in LUAD Cells

GSEA revealed the enrichment of cell cycle and apoptosis-related pathways in high-risk group (**Figures 5A**–**D**, **Table S4**). Therefore, we set to determine whether the anti-proliferative activity of gliclazide was due to effects on cell cycle and/or apoptosis. We treated A549 and H1299 cells with gliclazide for 48 h, and performed the flow cytometry for cell cycle analysis. Compared with the untreated cells, the G1 proportion of LUAD cells treated with gliclazide increased significantly (P <0.05), indicating that gliclazide treatment induced G1 cell cycle arrest (**Figure 8A**). Next, we investigated the effect of gliclazide on the protein levels of CyclinD1 and PARP, markers of cell cycle and apoptosis, respectively. Treatment with gliclazide for 72 h significantly decreased the CyclinD1 expression and increased the cleaved-PARP levels both LUAD cell lines (**Figure 8C**), suggesting that gliclazide could inhibit cell cycle progression and induce apoptosis in LUAD cells.

## Discussion

In the present study, we established 5-gene signature based on RS for the prediction of OS in LUAD patients, and identified Gliclazide as a drug candidate for treatment of LUAD with high-RS. Mechanistically, Gliclazide inhibited the proliferation of lung adenocarcinoma cells possibly by targeting CCNB1, CCNB1, CDK1 and AURKA to induce cell cycle arrest and apoptosis.

Accurate prognostic prediction and individualized clinical treatment strategy are the basis of precision medicine (11). Therefore, it is important to screen the potent prognostic biomarkers. To date, a large number of studies had been focused on the screening of prognostic biomarkers (26, 27). It was reported that SBP1 was significantly down-regulated in intrahepatic cholangiocarcinoma (ICC), which can be considered as a prognostic indicator or potential target for ICC therapy (26). For example, Xie et al. identified a six-gene prognostic model to predict the OS of NSCLC adenocarcinoma (9). However, these studies were rarely applied due to insufficient sample size, biological heterogeneity, diversity of expression platforms and lack of verification. Moreover, although Kratz et al. have screened out 14 risk-related genes in non-lung squamous cell carcinoma, and are performing clinical trials (27), we still noticed some limitations in the studies; 1) 14 genes were screened out from 217 prognostic risk genes derived from previous literature data, but not all genes from high-throughput sequencing data; 2) it is unknown whether the expression of these 14 genes between cancer tissues and normal tissues are different; 3) the signature more than 10 genes increased the complexity and difficulty of detection. Therefore, in order to improve the accuracy and robustness of the biomarkers, we performed comprehensive differential expression analyses on a large cohort of patients from three independent datasets with the same platform for the first time. Our study identified 5-gene signature, which was significantly related to the OS of LUAD patients and verified the gene signature in different datasets. We demonstrated the robust, reliable and stable prognostic prediction value of the 5-gene signature, thereby, providing a reliable prognostic tool for patients with LUAD. The strong correlation of 5-gene signature with clinicopathological factors, such as T-stage or N-stage, further supported the association of high-RS with progression and metastasis of LUAD.

Among the 5-gene signature identified in this study, it is known that KIF20A (Kinesin family member 20A) is involved in spindle formation during cell division (28), and Keratin 6A (KRT6A), a member of keratin proteins family, mainly lead to epidermalization of squamous epithelium. Both KIF20A and KRT6A were reported to be highly expressed in tumor tissues and associated with poor prognosis in multiple cancers, such as prostate, breast, gastric and pancreatic cancers (29–32). Moreover, they could also promote proliferation and migration, as well as inhibit apoptosis in lung cancer (33). KLF4, a member of SP/KLF transcription factor family, was reported to be down-regulated in gastric, colon, breast, lung cancers, and repress tumor proliferation and migration (34–38). Similarly, LIFR (leukemia inhibitory factor receptor alpha) was also reported to be down-regulated in LUAD and liver hepatocellular carcinoma, and inhibit local invasion and metastatic colonization in a variety of tumors (39, 40). RGS13, the smallest member of the RGS (G protein signal transduction regulator) family mainly expressing in B lymphocytes and mast cells (MC), was known to be related to immune-related diseases including human B lymphoma, allergic asthma and myasthenia gravis, and function as attenuating G protein mediated signal transduction (41, 42). However, its function in cancers was largely unknown. This study suggested that RGS13 might function as a suppressor gene in LUAD and this may be worthy of further validation in the future studies. In summary, except for RGS13, the role of the other four genes in cancer was all relatively exact, confirming the reliability and robustness of our 5-gene signature as a LUAD biomarker. In the future research, large-scale forward-looking investigation should be used to further evaluate the robustness of this signature.

Although much progress, such as target therapy and immunotherapy, has been made in lung cancer treatment, only part of patients could benefit from it (43). Therefore, it still needs to develop new drugs for lung cancer therapy. However, compared with the research and development of new drugs, the new use of old drugs was more cost-effective. Connectivity map (CMAP), which is a transcriptional expression database of human cancer cells treated with compounds or drugs, could be used for drug prediction based on the gene expression change in disease (14, 44). Chen et al. used CMAP and molecular docking to screen out Prestwick-685 and menadione as important new drug candidates for esophageal carcinoma (ESCA) (45, 46). In this study, by analyzing 94 survival-related DEGs of LUAD in CMAP, we identified Gliclazide as a potential therapeutic agent for the high-RS population of LUAD. Gliclazide is a sulfonylurea oral drug, which reduces the level of blood glucose by stimulating insulin secretion by islet β-cells. Because of its high safety and little side effects, gliclazide is widely used for the treatment of type 2 diabetes (47). It was reported that gliclazide could attenuate the toxic effect of reactive oxygen species in β cells by its antioxidant function, thus reducing the risk of complications caused by oxidative stress in patients with diabetes (48–51). Notably, several clinical trials also showed that cancer patients could benefit from gliclazide because of its antioxidant effect (48). However, the contradictory results were also reported in several studies. Piccinni et al. reported that the use of gliclazide could attenuate the efficiency of anti-cancer therapy in bladder cancer. It was also found that the antioxidant effect of gliclazide was able to protect apoptosis not only in normal cells but also in cancer cells (50); gliclazide could promote DNA repair in cancer cells rather than normal cells (48, 49). Therefore, the role of gliclazide in cancers remains quite unclear. In current study, we found that gliclazide could play anti-cancer role in LUAD cells. For the molecular mechanism investigation, using SwissTargetPrediction Online database and molecular docking, we identified CCNB1, CCNB2, CDK1 and AURKA as the key target candidates of Gliclazide. And the following WB analysis showed that both the G1 phase checkpoint CCND1, and G2-related proteins AURKA and CCNB1, were all down-regulated after gliclazide treatment. So, we considered that the function of gliclazide on G1 arrest might be stronger than its function on G2M arrest, thus leading to the final G1 phase arrest result by FACS detection. Certainly, this result needs to be confirmed in more kinds of cancer cells. As we known, the long-term cell cycle arrest could lead to apoptosis, which supported by our result that cleaved-PARP is up-regulated. Therefore, gliclazide plays its anti-cancer role by inducing cell cycle arrest and even apoptosis in LUAD cells.

At present, EGFR-tyrosine kinase receptor inhibitor (EGFR-TKI), a widely used drug in the clinical targeted therapy, had significantly improved the prognosis of NSCLC patients. However, only 20% of them with EGFR mutant can be benefit from it and therapies for patients without EGFR mutant (EGFR wild type) are still limited. It is necessary to develop new therapies for patients with EGFR wild type. Our study also focused on anti-cancer role of Gliclazide, thus H1299 and A549lung cancer cell lines, both of which are with wild type EGFR, were selected for experimental *in vitro*. Although the p53 status of two cells are different that wild type p53 in A549 but mutant p53 in H1299, we found that no matter the p53 status is wild type or mutant, gliclazide could induce G2M phase arrest and apoptosis in both of cell lines. Interestingly, the function of 5-gene signature is closely related to biological processes such as “Cell Cycle” and “DNA Replication”. These findings highlight the tight association of LUAD DEGs including the 5-gene signature with the processes of cell cycle and support the possible targeting of cell cycle regulate risk by gliclazide for the inhibition of high-risk LUAD patients. In our analysis, we found that the prognosis of patients with high-RS and low-RS was distinctly different, which suggested that active treatments are required for Stage I patients with high-RS rather than Stage II patients with low-RS. Based on these results, gliclazide has a great and promising therapeutic potential for NSCLC patients with high-RS.

Of note, relatively high dosage of gliclazide was required to inhibit LUAD cells, suggesting that its limitation for clinical application as a single drug. In fact, even for the anti-cancer drugs and PD-L1/PD-1 inhibitors, the effect of single drug on cancer therapy is not good enough. Two- or three-drugs combination is the common therapeutic regimens, but only part of tumor patients including lung cancer could benefit from them. Therefore, the combination of gliclazide and chemotherapy or targeted therapy, but not gliclazide alone, is more likely to achieve clinical application. On the other hand, drug modification combined with high-throughput screening is also a potential method for searching more efficient gliclazide derivatives for LUAD therapy (52). In this study, we preliminarily clarified its anti-tumor mechanism, and would explore the combined effect of gliclazide and chemotherapy or targeted drugs, and synthesize more gliclazide derivatives in future research work.

In summary, we identified 5-gene signature, constructed a risk score, and established nomogram based on 5-gene signature exhibiting powerful prognostic prediction effect for LUAD patients. For patients with high-RS, we found that gliclazide might be a promising anti-cancer drug by targeting cell cycle. The findings of this study provided an important reference for the prognosis and treatment of LUAD in terms of molecular biology and methodology, and thus are of great significance.

## Data Availability Statement

The original contributions presented in the study are included in the article/**Supplementary Material**. Further inquiries can be directed to the corresponding authors.

## Author Contributions

YCheng and XC conducted bioinformatics analysis and wrote the main manuscript. XC and XHu designed the entire project and supervised all experiments. YCheng and KH conducted all the experiments and analyzed the data. YW, YChen, JQ, BY, XZ, ST, DS, XH, XW, and YL provided bioinformatics analysis technology, experimental technology and writing support. All authors contributed to the article and approved the submitted version.

## Funding

Funding was provided by the National Natural Science Foundation of China (No. 81972197, No. 81472193), the Key Research and Development Program of Liaoning Province (2018225060) and the Technological Special Project of Liaoning Province of China (2019020176-JH1/103).

## Conflict of Interest

The authors declare that the research was conducted in the absence of any commercial or financial relationships that could be construed as a potential conflict of interest.

## Supplementary Material

Supplementary Figure 1 5 gene expression in TCGA -LUAD **(A–E)**; The association between5-gene signature and OS in subgroup of the TCGA-LUAD and GSE50081cohort. Kaplan-Meier survival curves were plotted to estimate the OS probabilities for the low-or high-RS group in the discovery group **(F–J)**, validation group GSE50081 **(K–R)**.

## Abbreviations

LUAD, lung adenocarcinoma; GEO, Gene Expression Omnibus; TCGA, The Cancer Genome Atlas; DEGs, Differentially Expressed Genes; CMAP, The Connectivity Map; RS, Risk Score; OS, overall survival; NSCLC, Non-small-cell lung cancer; TKIs, Tyrosine kinase inhibitors; DFS, Disease-free survival; GSEA, Gene Set Enrichment Analysis; MSigDB, Molecular Signatures Database; ROC, The Receiver Operating Characteristic; AUC, The area under the ROC curve; HR, hazard ratio; CI, confidence interval.
