# Supplementary figures and images for: Identification of Prognostic Signature and Gliclazide as Candidate Drugs in Lung Adenocarcinoma
Source: Front Oncol. 2021 Jun 24;11:665276. doi: 10.3389/fonc.2021.665276 (PMC8264429; doi:10.3389/fonc.2021.665276)

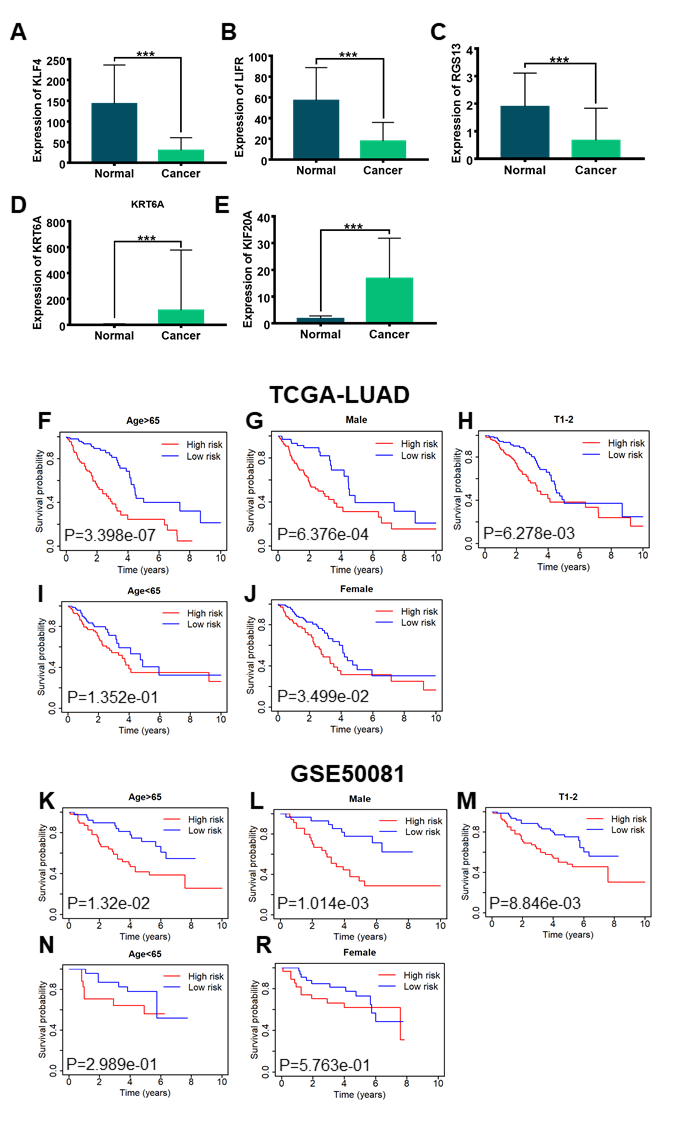

Supplement: Supplementary Figure 1 — 5 gene expression in TCGA -LUAD (A–E); The association between5-gene signature and OS in subgroup of the TCGA-LUAD and GSE50081cohort. Kaplan-Meier survival curves were plotted to estimate the OS probabilities for the low-or high-RS group in the discovery group (F–J), validation group GSE50081 (K–R). [file Image_1.tif]
