# Supplementary material for: Identification of Prognostic Signature and Gliclazide as Candidate Drugs in Lung Adenocarcinoma
Source: Front Oncol. 2021 Jun 24;11:665276. doi: 10.3389/fonc.2021.665276 (PMC8264429; doi:10.3389/fonc.2021.665276)
Supplement: Supplementary file 2 [file Table_1.docx]

Supplementary Material

# Supplementary Table1. Significant genes in Lasso regression analysis.

| Gene | Coef |
| --- | --- |
| ANLN | 0.10255715398085 |
| KIF20A | 0.0053266874581508 |
| LIFR | -0.00234713607658915 |
| KIF14 | 0.0654535932627844 |
| RGS13 | -0.0282781762432953 |
| KLF4 | 0.0514249391868795 |
| SLC7A11 | 0.0166796427858411 |
| CX3CR1 | -0.0237964798846535 |
| KRT6A | 0.0734960719041713 |

# Supplementary Table 2. Basic clinicopathologic features and correlation analysis of clinical parameters.

| Features | Numbers | Risk score | | |
| --- | --- | --- | --- | --- |
|  |  | Low expression  [example(%)] | High expression  [example(%)] | *P-value* |
| TCGA |  | | |  |
| Gender |  | | | 0.048 |
| Female | 170 | 94 (55.3) | 76 (44.7) |  |
| Male | 172 | 72 (44.4) | 90 (55.6) |  |
| Age (years) |  | | | 0.117 |
| ≥ 60 | 97 | 42 (43.3) | 55 (56.7) |  |
| < 60 | 235 | 124 (52.8) | 111 (47.2) |  |
| T-stage |  | | | 0.056 |
| T1 | 100 | 58 (58.0) | 42 (42.0) |  |
| T2-T4 | 232 | 108 (46.6) | 124 (53.4) |  |
| N-stage |  | | | 0.006 |
| N0 | 212 | 118 (55.7) | 94 (44.3) |  |
| N1-N3 | 210 | 48 (40.0) | 72 (60.0) |  |
| M-stage |  | | | 0.115 |
| M0 | 311 | 159 (51.1) | 152 (48.9) |  |
| M1 | 21 | 7 (33.3) | 14 (66.7) |  |
| GSE50081 |  |  |  |  |
| Gender |  |  |  | 0.216 |
| Female | 63 | 35 (55.6) | 28 (44.4) |  |
| Male | 65 | 29 (44.6) | 36 (55.4) |  |
| Age (years) |  |  |  | 0.804 |
| >= 60 | 19 | 10 (52.6) | 9 (47.4) |  |
| < 60 | 109 | 54 (54.5) | 55 (54.5) |  |
| T-stage |  |  |  | <0.001 |
| T1 | 43 | 32 (74.4) | 11 (25.6) |  |
| T2-T4 | 85 | 32 (37.6) | 53 (62.4) |  |
| N-stage |  |  |  | 0.689 |
| N0 | 94 | 48 (51.1) | 46 (48.9) |  |
| N1 | 34 | 16 (47.1) | 18 (52.9) |  |
|  |  |  |  |  |

# Supplementary Table 3. Univariate and multivariate Cox analysis of the five- gene signature and clinical risk factors.

| Variables | Univariate analysis | | Multivariate analysis | |
| --- | --- | --- | --- | --- |
|  | HR (95% CI) | P-value | HR (95% CI) | P-value |
| TCGA |  |  |  |  |
| Age (years) | 1.002(0.985-1.021) | 0.793 | 1.021(1.002-1.041) | 0.029 |
| Gender (male vs. female) | 1.114(0.793-1.564) | 0.534 | 0.864(0.610-1.225) | 0.412 |
| T-stage (T2&T3&T4 vs. T1) | 2.015(1.295-3.136) | 0.002 | 1.417(0.894-2.246) | 0.138 |
| N-stage (N1&N2&N3 vs. N0) | 2.494(1.772-3.510) | <0.001 | 2.189(1.528-3.137) | <0.001 |
| M stage (M1 vs. M0) | 1.854(1.044-3.294) | 0.035 | 1.974(1.094-3.560) | 0.024 |
| Risk score | 1.658(1.464-1.877) | <0.001 | 1.591(1.391-1.821) | <0.001 |
| GSE50081 |  |  |  |  |
| Age (years) | 1.020(0.991-1.050) | 0.174 | 1.012(0.982-1.042) | 0.446 |
| Gender (male vs. female) | 1.344(0.775-2.330) | 0.293 | 1.393(0.797-2.437) | 0.245 |
| T stage (T2&T3&T4 vs. T1) | 2.592(1.299-5.173) | 0.007 | 1.664(0.775-3.575) | 0.192 |
| N stage (N1 vs. N0) | 2.241(1.266-3.965) | 0.006 | 2.009(1.123-3.594) | 0.019 |
| Risk score | 4.494(1.740-11.605) | 0.002 | 3.003(1.113-8.106) | 0.030 |

# Supplementary Table 4 . The 18 significant enriched KEGG pathways in the TCGA cohort.

| NAME | NOM p-val | FDR q-val |
| --- | --- | --- |
| KEGG_CELL_CYCLE | 0 | 0.002 |
| KEGG_OOCYTE_MEIOSIS | 0 | 0.003 |
| KEGG_SPLICEOSOME | 0.008 | 0.015 |
| KEGG_PYRIMIDINE_METABOLISM | 0 | 0.025 |
| KEGG_DNA_REPLICATION | 0 | 0.024 |
| KEGG_MISMATCH_REPAIR | 0 | 0.032 |
| KEGG_NUCLEOTIDE_EXCISION_REPAIR | 0.002 | 0.036 |
| KEGG_P53_SIGNALING_PATHWAY | 0 | 0.045 |
| KEGG_HOMOLOGOUS_RECOMBINATION | 0.002 | 0.052 |
| KEGG_PROGESTERONE_MEDIATED_OOCYTE_MATURATION | 0.006 | 0.057 |
| KEGG_BASE_EXCISION_REPAIR | 0.008 | 0.061 |
| KEGG_CITRATE_CYCLE_TCA_CYCLE | 0.044 | 0.063 |
| KEGG_PROTEASOME | 0.022 | 0.062 |
| KEGG_RNA_DEGRADATION | 0.032 | 0.129 |
| KEGG_PENTOSE_PHOSPHATE_PATHWAY | 0.022 | 0.141 |
| KEGG_SMALL_CELL_LUNG_CANCER | 0.024 | 0.148 |
| KEGG_CYSTEINE_AND_METHIONINE_METABOLISM | 0.027 | 0.186 |
| KEGG_UBIQUITIN_MEDIATED_PROTEOLYSIS | 0.057 | 0.217 |

**
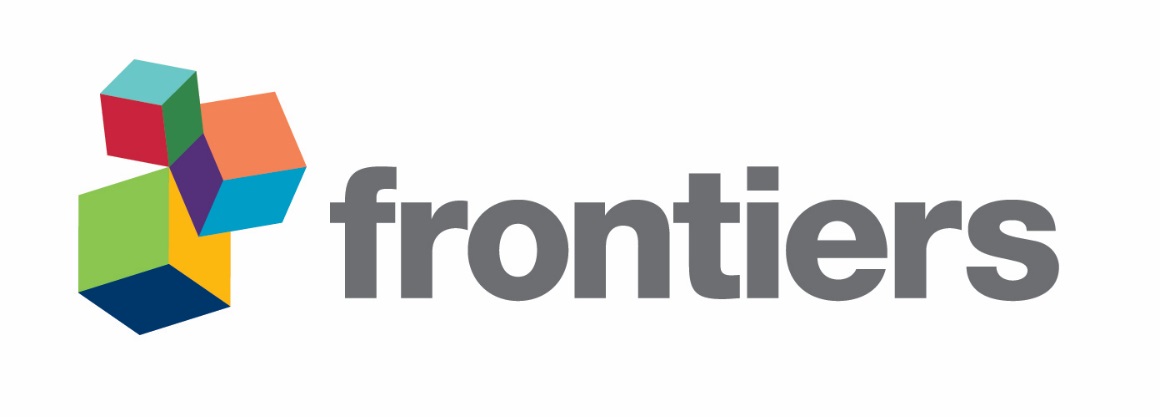
**
